# The epigenetic regulatory mechanism of PIWI/piRNAs in human cancers

**DOI:** 10.1186/s12943-023-01749-3

**Published:** 2023-03-07

**Authors:** Qun Zhang, Yazhi Zhu, Xinyu Cao, Wenhui Tan, Jianglong Yu, Yaqiong Lu, Ran Kang, Xiaolan Wang, Ermao Li

**Affiliations:** 1 Hengyang Medical School, University of South China, Hengyang, 421001 Hunan China; 2 Chuanshan College University of South China, Hengyang, 421001 Hunan China; 3 Department of Urology, Hengyang Medical School, The First Affiliated Hospital, University of South China, Hengyang, 421001 Hunan China; 4 Department of Reproductive Medicine, Hengyang Medical School, The First Affiliated Hospital, University of South China, Hengyang, 421001 Hunan China; 5 Institute of Translational Medicine, Hengyang Medical School, University of South China, Hengyang, 421001 Hunan China

**Keywords:** PIWI protein, piRNA, m^6^A methylation, DNA methylation, Histone modification

## Abstract

PIWI proteins have a strong correlation with PIWI-interacting RNAs (piRNAs), which are significant in development and reproduction of organisms. Recently, emerging evidences have indicated that apart from the reproductive function, PIWI/piRNAs with abnormal expression, also involve greatly in varieties of human cancers. Moreover, human PIWI proteins are usually expressed only in germ cells and hardly in somatic cells, so the abnormal expression of PIWI proteins in different types of cancer offer a promising opportunity for precision medicine. In this review, we discussed current researches about the biogenesis of piRNA, its epigenetic regulatory mechanisms in human cancers, such as N6-methyladenosine (m^6^A) methylation, histone modifications, DNA methylation and RNA interference, providing novel insights into the markers for clinical diagnosis, treatment and prognosis in human cancers.

## Introduction

PIWI subfamily proteins belong to the PAZ-PIWI domain (PPD) protein family, which is crucial for the biogenesis and function of small non-coding RNAs (sncRNAs) [1]. PPD family proteins are highly conserved and contain a relatively variable N-terminal domain, PAZ and MID domains located in the middle, and C-terminal PIWI domain [2–5]. The PAZ and MID domains can bind to some sncRNAs, such as microRNA (miRNA), small interfering RNA (siRNA) and PIWI-interacting RNA (piRNA) [2, 6, 7]. While the PIWI domain has similar function to RNase H, and can cut the target RNA through sncRNAs [8]. Argonaute(AGO) proteins, another subfamily of the PPD protein family, are ubiquitously present in diverse tissues [9]. They bind to well-known small RNAs (miRNAs, siRNAs) to perform gene regulatory functions mainly in the form of RNA-induced silencing complexes (RISCs) [10]. In turn, the PIWI protein mainly binds to piRNA and performs physiological functions in germ cells. In addition to the function of directly cutting and degrading target RNA similar to Ago protein/miRNA, PIWI protein/piRNA can also exert epigenetic regulatory functions through the recruitment of other epigenetic regulatory factors (such as DNA methylase, RNA methylase, deadenylyase, phosphorylation, etc.), which will be detailed below [11].

In human, PIWI family proteins consist of four members: PIWIL1 (HIWI), PIWIL2 (HILI), PIWIL3 (HIWI3) and PIWIL4 (HIWI2); and three in mice, as they are MIWI (Piwil1), MILI (Piwil2) and MIWI2 (Piwil4); and also three PIWI encoding-genes in drosophila including Piwi, Ago3 and Aub [12–15]. PIWI proteins are mainly expressed in germ cells, and barely found in normal somatic cells, exhibiting highly-conserved functions in varieties of species [13, 16, 17]. However, recent studies have found that PIWI proteins are also abnormally expressed in human tumors [18, 19]. Therefore, it is expected to be an excellent target for tumor targeted therapy. Because of this characteristic, PIWI proteins have attracted much attention in recent years. To elucidate the regulatory mechanism of PIWI proteins in cancer have become an important topic in cancer prevention and treatment research [17, 20–23].

### Overview of piRNAs

**Fig. 1:**
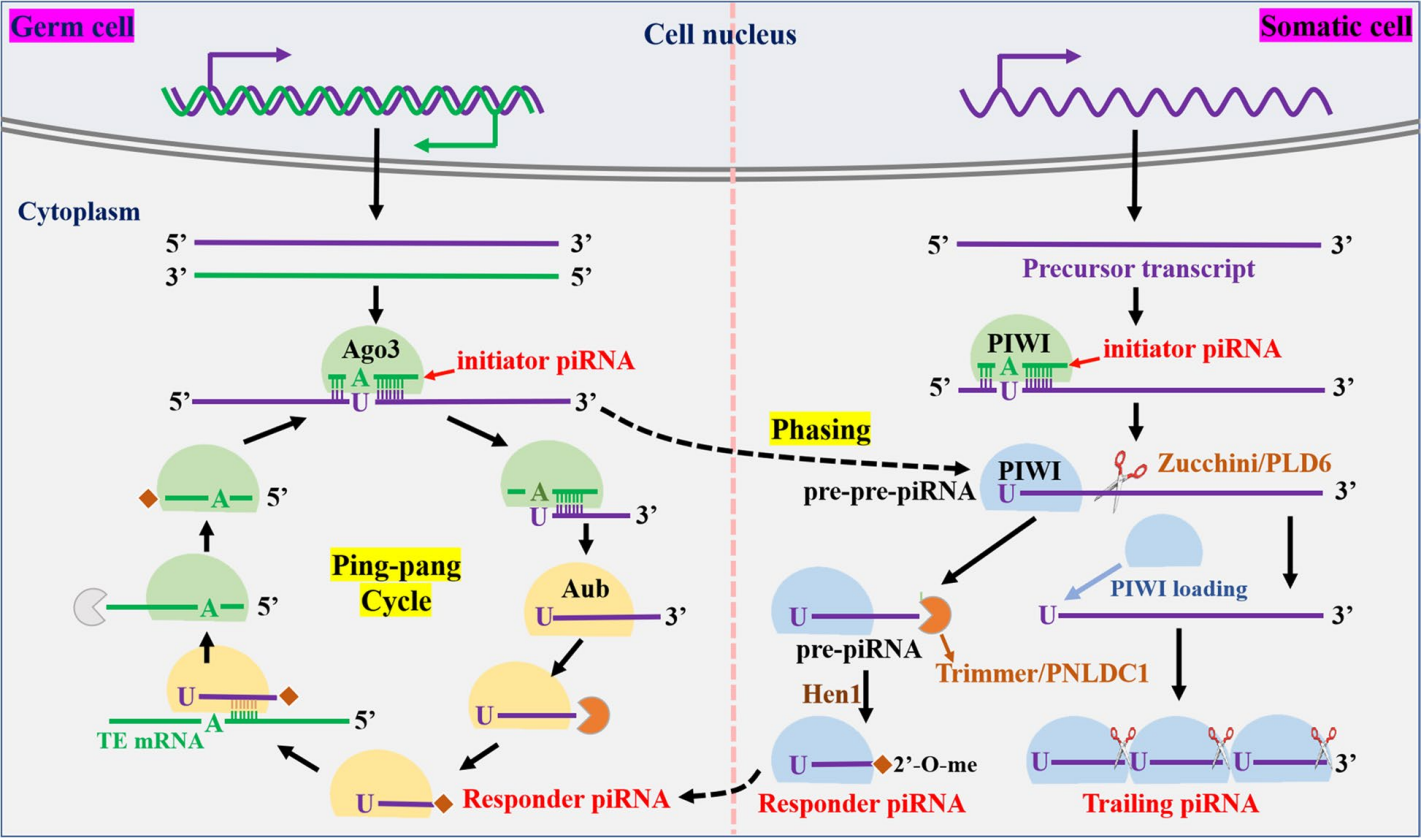
Two modes of piRNA production. In somatic cell, piRNA is mainly produced by Phasing processing pathways (Left part). First, piRNA precursor transcript is cleaved by PIWI/initiator piRNA to generate pre-pre piRNA, pre-pre piRNA binds to a new PIWI protein and is then cleaved into two parts by endonucerase (Zucchini in Drosophila or PLD6 in mice). The binding part of PIWI protein is the known intermediate piRNA (pre-piRNA), which generates mature response piRNA under the action of exonucerase (Trimmer/PNLDC1) and methyltransferase (Hen1). The remainder of the pre-pre piRNA is repeatedly bound by the PIWI protein at its 5' end and repeatedly cleaved by the Zucchini/PLD6 to produce a string of Trailing piRNA. In germ cell, piRNA is produced by the mutual cleavage of complementary transcripts from transposon and piRNA clusters mediated by Ago3 and Aub, called the “Ping-pang” cycle (Right part), resulting in pairs of piRNAs (“initiator” and “responder” piRNA) with a 10 base overlap at the 5’- end

PIWI proteins usually combine with piRNAs to exert gene regulation function, playing an important role in maintaining the stability and integrity of germ cell genome [24]. piRNAs, the largest subclass of sncRNAs, with a length of about 24-32nt and a 2’-O-methylated 3’-end, are firstly indicated to exist in the germline of the Drosophila, and gradually discovered in multiple species such as invertebrates and vertebrates [19, 22, 25–28]. Approximately 90% of piRNAs are produced from specific gene loci called piRNA clusters, which contain transposable element [29–31]. However, recent studies have also found that many piRNA sequences are derived from non-transposable element sequences, which mean that the function of piRNA is not only to silence transposable elements [32]. piRNA clusters are classified into double-stranded and single-stranded clusters according to their ability to produce piRNA from two or one strand on the genome [33, 34]. Single-strand piRNA cluster is the most widely distributed and default type, and its transcription is similar to the classical transcription pathway, with primary transcript also spliced normally [35]. Double-stranded piRNA clusters are mostly distributed in germ cells and lack well-defined promoter regions, newly generated piRNA transcripts cannot be splicing and polyadenylation [36]. The newly generated precursor transcripts are transported from the nucleus to the cytoplasm where they are further processed into mature piRNAs in a Dicer-independent manner, but this process is complex and not yet fully understood [36]. Currently, two interrelated mechanisms are known for model design: the primary amplification pathways (also called Phasing processing pathways) and the “Ping-Pong” amplification pathways, as shown in Fig. 1.

Phasing processing pathways are the main means of piRNAs generation in somatic cells [37]. Firstly, an initiator piRNA binding PIWI protein cuts the precursor transcript to form 5’-monophosphate substrate segments, which are called pre-pre-piRNA [38]. The pre-pre-piRNA binds to the corresponding PIWI protein and is subsequently cleaved by endonuclease (Zucchini in Drosophila or PLD6 in mice) [38–40]. The resulting 5’cleaved fragments are known as pre-piRNA, the 3-terminal of which can be further modified and cleaved by exonuclease (Trimmer/PNLDC1) and catalyzed by methyltransferase (Hen1) into 2’-O-methylation, thus becoming responder piRNA [39, 41]. Meanwhile, the 3’-cleavage fragments of pre-pre-piRNA binds to the next PIWI protein as a new pre-pre-piRNA and is cleaved again to produce a new PIWI binding pre-piRNA [36, 42]. Then, after processing by nuclease and methyltransferase at the 3’-end, it became mature trailing piRNA, and the 3’ cut fragment obtained this time is also introduced into the next PIWI protein as a new pre-pre-piRNA [43]. Since then, a series of trailing piRNA are continuously generated downstream of the responder piRNA [44, 45].

In germ cells, the responder piRNA produced by the primary amplification pathways continues to amplify in the “Ping-Pong” amplification pathway [46]. This amplification pattern accompanied by the intercutting of complementary transcripts from transposons and piRNA clusters, and is the main source of piRNA in germ cells [46]. The “Ping-Pong” cycle produces pairs of piRNAs (initiator piRNAs and responder piRNAs) with a 10-base overlap at the 5-end [47, 48]. Specifically, the “Ping-Pong” cycle is started by maternal AGO3-initiator piRNA, which identifies complementary piRNA precursors and cleaves them at nucleotide sites 10 corresponding to the 5’ end of the guide piRNA to generate pre-pre-piRNA [46–48]. Similarly, Aub protein binds to pre-pre-piRNA and is subsequently cleaved by endonuclease to form an intermediate piRNA (pre-piRNA), which is further processed into mature response piRNA under the action of 3' to 5' exonuclease and methyltransferase [49, 50]. The response piRNA is then used as a new initiator piRNA to guide Aub protein to bind to the long precursor transcript that is complementary to the response piRNA sequence [48, 51]. Aub protein cleaves the long precursor transcript to obtain the new pre-pre-piRNA [52]. The new pre-pre-piRNA is bound to a new AGO3 protein and cleaves to obtain the new pre-piRNA [52, 53]. The new pre-piRNA is pruned by exonuclease at the 3’ end and modified by 2’-O-methylation to obtain the new mature piRNA [53, 54]. This piRNA sequence is consistent with the initiator piRNA sequence at the beginning of the “Ping-Pong” amplification pathway, thus completing one round of “Ping-Pong” cycle [53]. Organisms generate mature piRNAs through these two routes. However, the Phasing processing pathway increases piRNA diversity by generating new piRNA, while the Ping-pong cycle pathway increases the abundance of existing piRNA [44].

### Regulation mechanisms of PIWI/piRNAs in normal germ cells and germline tumors

**Fig. 2:**
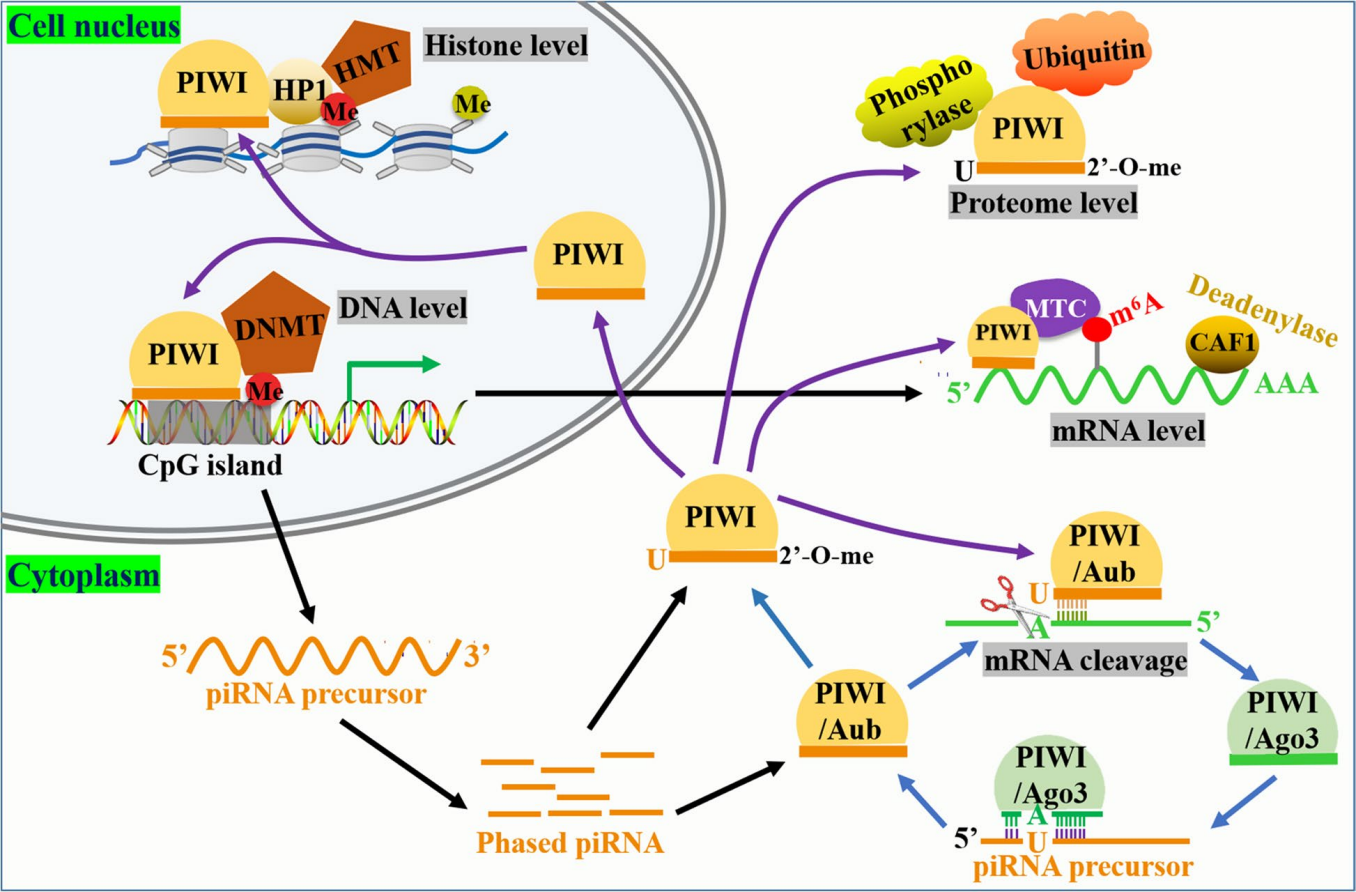
Regulation mechanism of PIWI-piRNA complex in normal cells. In normal cells, PIWI proteins mainly bind to piRNA to form complexes that regulate gene expression at the transcriptional or post-transcriptional level. At the transcription level, the target gene transcription is regulated mainly by nuclear localization PIWI protein recruiting histones and DNA modification enzymes. At the post-transcriptional level, in addition to the epigenetic regulatory factors such as MTC, ubiquitination enzyme and phosphorylase recruited by PIWI proteins in the cytoplasm and regulated at the level of RNA and proteasome, the target gene mRNA can also be directly targeted and cleaved in a PIWI endonuclease-dependent manner, which is realized in the “Ping-pong” cycle pathway

The most well-known and classical functions of PIWI/piRNAs complex is involvement in the regulation of transposon silencing and reproductive development, and its mechanism of action is to regulate gene expression at the transcriptional or post-transcriptional level (Fig. 2) [12, 44, 55]. The Transcription level regulation is mainly performed by nuclear located PIWI proteins (such as Drosophila Piwi and mouse MIWI2) [12, 26]. PIWI proteins entered the nucleus after they are loaded with piRNAs [56]. PIWI/piRNA complexes regulates target genes at the transcriptional level by recruiting epigenetic modification factors (e.g., histone modification enzymes, DNA methylases) [56]. This often occurs when the PIWI protein has low catalytic activity [57–59]. Disruption of the PIWI/piRNA signaling pathway or de novo DNA methylation can lead to male sterility [60–62]. De novo DNA methylation is an important physiological process in epigenetic. It refers to the methylation of cytosine C5 in DNA strands at a new site without dependence on existing methylated DNA strands [63]. A team from the University of Edinburgh found that piRNAs can bring MIWI2 to the adjacent transposition element transcription, inhibit chromatin remodeling activity and activate de novo methylation protein function through MIWI2 interaction with SPOCD1, suggesting that piRNA is an important actor directing de novo DNA methylation [64]. Post-transcriptional gene regulation is usually performed by PIWI proteins in the cytoplasm, such as Drosophila Aub and Ago3, mouse MIWI and MILI, which dependent on or independent of the endonuclease activity of PIWI proteins [52]. In endonuclease dependent mode, piRNA binds to PIWI protein to form RISCs complex, which is targeted to cut transposable element mRNA according to base complementary pairing principle [56]. At this point, piRNA is similar to miRNA [52]. In addition to silencing transposable element, PIWI/piRNA can also silence other mRNAs, pre-mRNAs, and lncRNAs at the post-transcriptional level [58, 65, 66]. In endonuclease independent mode, the piRNA/PAZ domain can bind other functional proteins, such as m^6^A methyltransferase complex (MTC) [67], deadenylase [68], phosphorylase [69] and ubiquitination enzyme [70], to regulate gene expression at the RNA and proteome level. These findings extend the diversity of piRNA regulated protein-coding genes, but the mechanisms behind these regulations are still not fully understood. The regulation of coding genes depends on the level of base complementary pairing between target miRNA and piRNA [71]. For example, cutting requires higher complementarity [72]. However, in the non-cutting condition, PIWI protein needs to recruit different cofactors to achieve different regulatory effects on mRNA.

As mentioned above, PIWI/piRNA complex mainly plays a role in silencing transposons and regulating reproductive development in germ cells. Transposon silencing is generally achieved by inhibiting the expression of retrotransposons at both the transcriptional and post-transcriptional levels. Therefore, PIWI protein/piRNA deletion or epigenetic disruption may lead to the recovery of transposon activity, which is associated with germ cell tumors development [73] In contrast to normal testis, most piRNA are downregulated or completely lost in testicular germ cell tumor (TGCT) [74]. Ferreira et al. also found that the expressions of PIWIL1, PIWIL2 and PIWIL4 in both seminoma and non-seminoma were lower than those in normal testis [75]. More importantly, epigenetic inactivation of PIWI proteins lead to decreased piRNA production and hypomethylation of the retrotransposon, LINE-1, whose activation can affect the expression or regulation of other genes in the genome, resulting in germ cell tumors due to effects on genomic stability [76]. In short, PIWI protein/piRNA loss makes cells lose the role of silencing transposons in germ cell tumors, and the activation of retrotransposon is an important mechanism of germ cell tumor occurrence.

**Fig. 3:**
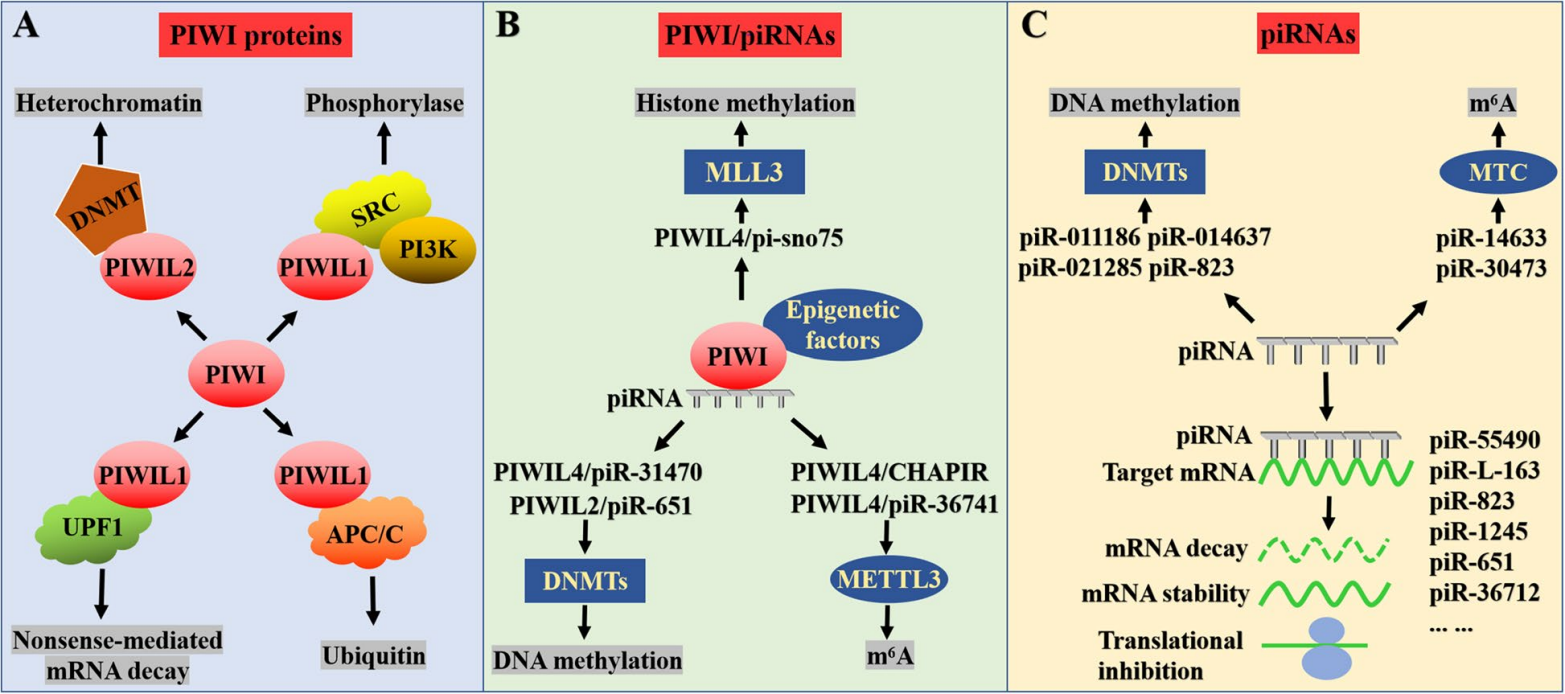
The epigenetic regulatory mechanism of PIWI/piRNAs on human cancers. **A** PIWI proteins regulate gene expression in cancer in a piRNA independent manner, mainly through binding with other functional proteins (such as methyltransferase, ubiquitination enzyme, phosphorylase, etc.); **B** PIWI proteins regulate gene expression in cancer in a piRNA dependent manner, which is similar to that in normal cells; **C** piRNAs independently regulate mechanisms in cancer, exerting epigenetic regulatory functions by binding with epigenetic regulatory factors or directly regulating their expression. piRNAs can also bind with target gene to degrade mRNA, change its mRNA stability, or inhibit its translation process

Unlike in germ cell tumors, PIWI proteins have been reported to be highly expressed in a variety of tumors, such as gastric, colon, liver, glioma, and bladder cancer [77]. Furthermore, the study of piRNA is still in the preliminary stage, and its epigenetic regulation mechanism in tumors is still under investigation. In this review, we summarize the up-to-date information about the epigenetic regulatory mechanisms of PIWI/piRNAs in human cancers, ranging from m^6^A methylation, histone modifications to DNA methylation and others (Fig. 3), which produces unique observations of biomarkers for cancer diagnosis and prognosis, together with treatment.

## Epigenetic regulatory mechanism of PIWI/piRNAs in human cancers

### m^6^A RNA methylation

In recent years, m^6^A, one of the most popular epigenetic RNA modifications, has been reported that it plays a vital role in modulating gene expression. This RNA modification can be realized by m^6^A “writers”, “erasers” and “readers” and these proteins can, respectively, methylate, demethylate and identify m^6^A on RNA. The MTC are composed of at least seven “writer” proteins, METTL3, METTL14, METTL16 [78], WTAP, RBM15/15B [79], KIAA1429 [80], ZC3H13 [81]. Currently, there are mainly two types of m^6^A demethylases: FTO and ALKBH5. Their discovery suggests that m^6^A methylation is a reversible modification just like DNA and histone modification [82]. To perform a specific biological function, m^6^A must be recognized by a specific RNA-binding protein. These methylated reading proteins, such as YT521-B homology (YTH) domain proteins, the heterogeneous nuclear ribonucleoprotein (HNRNP), eukaryotic translation initiation factor 3 (elF3) and the IGF2 mRNA binding protein (IGF2BP) family, affect the fate of mRNA in an m^6^A-dependent manner, including RNA transcription, processing, translation and metabolism [83].

Moreover, increasing evidences indicate that m^6^A RNA methylation also potentially influences the tumorigenesis. Therefore, some people wonder whether there exist some relations between piRNA and m^6^A that can impact on the cancer progression together. And here are some related researches as followed. Xie’s research shows that piRNA-14633 can facilitate the malignancy in cervical cancer (CC) by piRNA-14633/METTL14/CYP1B1 axis [84]. In this study, piRNA-14633 is highly expressed in CC cells, and it raises m^6^A RNA methylation levels and METTL14 mRNA stability. Also, METTL14 regulated by piRNA-14633 may target at CYP1B1 in CC cells, and thus promoting the proliferation, migration and invasion capacity of CC cells. Han’s study presents the fact that piRNA-30473 plays a role in diffuse large B-cell lymphoma (DLBCL) by regulating WTAP [85]. Similarly, as a negative prognosis index in DLBCL patients, piRNA-30473 is overexpressed in DLBCL, and it regulates m^6^A methylation level by regulating the expression of WTAP, together with IGF2BP2, one of the m^6^A readers, which can up-regulate the expression of HK2 targeted at 5’UTR of its mRNA in cells by increasing stability of HK2 mRNA. Both studies show that piRNAs perform the role of m^6^A modification by regulating the expression of m^6^A methyltransferase.

Besides, researchs have also found that piRNAs can regulate the global cellular m^6^A level through interacting with some m^6^A methyltransferase and affecting its activity. For instance, Liu’s founds that piRNA-36741 regulates osteoblast differentiation by controlling METTL3-dependent m^6^A methylation of BMP2 [86]. CHAPIR (cardiac-hypertrophy-associated piRNA)-PIWIL4 complex fosters pathological hypertrophic and remodels heart by interacting with METTL3 and prohibiting the m^6^A methylation of Parp10 mRNA transcripts to up-regulate PARP10 expression, and thus the accumulation of NFATC4 in nuclear [67]. Mechanistically, both studies conclude that the piRNA-PIWIL4 complex directly interacted with METTL3 and regulated METTL3-mediated m^6^A modification of targeting gene transcripts.

Although such studies prove that piRNA-PIWI protein complex can occupy the useful role of m^6^A modification by regulating the expression of m^6^A methyltransferase or influencing its m^6^A activity, several crucial issues remain unresolved. For example, piRNA can recruit DNA methyltransferase through PIWI protein to regulate DNA methylation of target gene, similarly with miRNA, piRNA as a ncRNAs binding to 3’UTR or CDS region of mRNA. Accordingly, whether some piRNA only regulate m^6^A level of target gene binding to it, just like DNA methylation, rather than the global cellular m^6^A level. Furthermore, studying this issue in-depth may also be expected to clarify the molecular mechanism about how m^6^A is specifically and dynamically deposited in the transcriptome [87]. Obviously, there are only a few studies have been done. The regulatory mechanism of piRNA, together with m^6^A, enjoys immense potential in human cancers. Further researches should be carried out to dig into the deep internal mechanisms.

### Histone modification

Nucleosomes, the basic unit of chromosome function, are formed by 146 pairs of bases surrounding a histone octamer consisting of two molecules each of four histone proteins (H2A, H2B, H3 and H4). Some histones of eukaryotes are able to stabilized nucleosome, while the free amino acid residues at the terminal of histone can undergo covalent modification such as methylation, acetylation, phosphorylation, ubiquitination and adenosine diphosphate ribosylation and glycosylation [88], among which methylation and acetylation are two important modification. Histone acetylation is a reversible dynamic equilibrium process mediated by histone acetyltransferase (HAT) and histone deacetylase (HDAC). HAT can transfer the acetyl group carried by acetyl-CoA to the specified lysine residue at the N terminal of histone, and the acetyl group neutralize the positive charge carried by the residue, so as to expand the DNA conformation and relax the nucleosome structure, thus promoting the binding of DNA with transcription factors and activating the transcription of specific genes [89]. Histone methylation usually occurs on lysine and arginine residues, methylation at different sites has different effects on histone function [90]. These modification enzymes can be divided into two parts, one is the activation marker, the other is repression marker. Histone H3 lysine 4 trimethylation (H3K4me3), H3 and H4 acetylation, and histone H3 lysine 36 (H3K36me2 and H3K36me3) are activation markers, which is beneficial to nucleosome’s formation of three-dimension structure, as access for transcription of open chromatin [51]. Repressive markers, such as trimethylation of histone H3 lysine 9 and 27 (H3K9me3 and H3K27me3), in the contrast, are able to generate a condensed chromatin [91].

Different kinds of piRNA can recruit corresponding modified enzymes of histones, and thus resulting in different effects. For example, one research shows that pi-sno75 can up-regulate the expression of pro-apoptotic protein TRAIL and play a inhibitory role in breast cancer. Mechanistically, pi-sno75 binds to PIWIL1/4 and induces H3K4 methylation/H3K27 demethylation of TRAIL by recruiting MLL3/hCOMPASS complex [92]. Wu et al. demonstrate that CDKN2B-related piRNAs, hsa-piR-011186 and hsa-piR-014637 were highly expressed in leukemia cells U937, and their elevated expression could inhibit CDKN2B expression, promote cell cycle progression and induce apoptosis [93]. Mechanistically, hsa-piR-011186 and hsa-piR-014637 form complexes with DNMT1, Suv39H1 and EZH2 proteins that regulate the methylation level of the DNA and Histone H3 in the CDKN2B promoter site. This unique piRNA complex speed up epigenetic modifications about the cell cycle, which provides new insights into the advancement of leukemia. Another research indicates that there exists a strong correlation between the low-expressed of PIWIL2 in invasive breast carcinomas (IBCs) and the downregulation of DNMT1, histone H1, HP1 and SUV39H1, which are involved in chromatin accessibility and genome methylation [94]. PIWI /piRNA dysfunction, which leads to DNA demethylation and reactivation of transposition elements, is an epigenetic mechanism underlying changes in the genome integrity and immune response of many tumor cells. Moreover, PIWIL2 underexpression is greatly related with increased immune cytotoxic CD8 + response in IBCs, it demonstrates the feasibility of being predictive biomarkers for immunotherapies, and the possible novel clinical diagnostic markers.

### DNA methylation

DNA methylation refers to the process that organisms transfer methyl to a specific base with S-adenosine methionine (SAM) as the methyl donor under the catalysis of DNA methyltransferases (DNMTs), and is critical in silencing gene on the long-term basis, specifically in the regions of promoter [95, 96]. In mammals, DNA methylation occurs primarily on the fifth carbon atom of cytosine in the CpG island, called 5-methylcytosine (m5C) [97]. CpG islands in normal cells are demethylated due to protection, genome-wide hypomethylation, uncontrolled regulation of enzymes that maintain methylation patterns, and hypermethylation of normal unmethylated CpG islands are common phenomena in human tumors [98–101], previous studies have shown that hypermethylation of promoter region leads to inactivation of tumor suppressor genes, which is one of the common characteristics of human tumors [91, 102]. As known to all, lncRNA can recruit DNMTs to improve the DNA methylation level [103]. Most importantly, recent studies indicate that piRNAs can also affect the hypermethylation by recruiting DNMT in some human cancers [104, 105]. The interaction between DNA methylation and piRNAs exerts deep influence on the stability and expression of genome, eventually resulting in the abnormal change of the cell signaling, and thus getting the diseases moving ahead [106, 107].

Many studies have found that the changes of DNA methylation in tumor cells are closely related to PIWI/piRNA disorders. In breast cancer, piR-651 [105], piR-823 [108] and piR-021285 [109] is highly expressed compared with normal breast tissue. However, the expressions of PIWI proteins in breast cancer have not been unified. Didier et al. found that PIWIL2 was expressed in normal breast tissue but not in tumor tissue [94], whereas three other studies showed the opposite [105, 110]. The overexpression of piR-651 can promote the proliferation and invasion of breast cancer cells, and its regulatory mechanism is through recruiting DNMT1 to the promoter region of the tumor suppressor gene PTEN through PIWIL2, and the PTEN promoter is methylated, thus reducing its expression level [105]. Also, Fu et al. demonstrated that piR-021285 can promote the methylation of proto-oncogene ARHGAP11A 5’UTR/first exon and down-regulate its expression level, but its specific regulatory mechanism needs further study [109]. Ding et al. found that the overexpression of piRNA-823 promotes the expression of DNMTs, such as DNMT1, DNMT3A and DNMT3B, and thus inducing the hypermethylation of gene adenomatous polyposis coli (APC), inducing the stemness of luminal breast cancer cells by triggering Wnt signaling pathway [108]. In addition to breast cancer, piR-823 was also found to be upregulated in multiple myeloma (MM) and esophageal squamous cell carcinoma (ESCC) [104, 111, 112].

The overexpression of piR-823 in MM can maintain the stemness of multiple myeloma stem cells and increase the tumorigenic potential by activating DNMT3B and upregating DNA methylation level [112]. Analogously, Yan et al. shows that piRNA-823 can up-regulate both mRNA and protein levels of DNMT3A and 3B, leading to the methylation of tumor suppressor gene p16(INK4A), thus playing a cancer-promoting role in MM [113]. Also, Su et al. shows that both piR-823 and DNMT3B are overexpressed in ESCC and they have positive relations with each other [107]. piR-823 may promote ESCC progression through DNMT3b-mediated DNA methylation.

PIWI/piRNA mediated DNA methylation also plays an important role in other tumors. In prostate cancer, highly expressed piRNA-31470 directs DNMT1 and DNMT3α to bind to CpG islands of glutathione S-transferase pi 1 (GSTP1) via PIWIL4. Subsequently, GSTP1 is methylated, its transcription is inhibited, and the inactivation of GSTP1 increases the sensitivity of normal cells to oxidative stress and the risk of prostate cancer [114]. Wu’s study indicates that the overexpression of LV-has-piR-011186 binding with a particular sequence recruits DNA and histone-methylating proteins to the CDKN2 promoter gene, leading to the result of the promotion of cell proliferation, as well as the inhibition of cell apoptosis in U937 leukemia cells [93]. In lung cancer, RASSF1C, a major member of the RASSF1 gene family [115, 116], can encourage the expression of PIWIL1, as well as the modulation of piRNA’s expression [117]. This research presents us the RASSF1C/PIWIL1/piRNA axis, modulating the Gem Interacting Protein (GMIP) mRNA expression by DNA methylation in lung cancer, and thus affecting the migration of cancer cells.

### Other oncogenic mechanisms of PIWI/piRNAs

As mentioned before, in the germ cells, PIWI protein can cleave the transposon RNA that is complementary to a piRNA, dependent on its splicing enzyme activity, which leads to transposon gene post-transcriptional silencing, which is related to piRNA biosynthesis [118]. However, this condition seems to be different in cancer. According to the researches at present, PIWI protein and piRNA seem to regulate mRNA decay as two independent individuals rather than as a whole (Fig. 2). For example, Liu et al. found that PIWIL1, as a coactivator of APC/C complex rather than a substrate, targets cell adhesion protein Pinin for proteolytic ubiquitination in the absence of piRNA, thus promoting pancreatic cancer metastasis [119]. Lin et al. also proves that piRNA expression could hardly be detected in gastric cancer cells with high expression of PIWIL1, abolishing the piRNA-binding activity of PIWIL1 can still play the biological functions of promoting cancer cell growth and tumor metastasis [7]. Mechanistically, PIWIL1 is likely to negatively regulate the expression of tumor suppressor genes by binding protein, a core factors of nonsense-mediated mRNA decay machinery. It seems that PIWI protein can directly bind to some functional proteins (ubiquitinase and phosphorylase) to play an epigenetic regulatory function in tumor independent of piRNA. Whether this phenomenon also exists in normal cells needs further study. Similarly, many piRNAs can regulate post-transcriptional networks to inhibit target function through piRNA-RNA interactions in cancer, similar to miRNA mechanisms [120–122]. In lung cancer cells, piRNA can regulate the growth, apoptosis, migration and invasion of cancer cells through P-ERM, Caspase-3 and Akt/mTOR [123–125]. In colon cancer cells, piRNA regulates the growth apoptosis and cell cycle operation of cancer cells through HSF1, BTG1 and FAS [69, 126, 127]. Only PWIL1 protein is expressed in human colon cancer COLO205 cells, while other PIWI proteins are not expressed [17]. In the Head and neck squamous cell carcinoma related to hpv16/18, the depletion of piRNA FR140858 can possibly boost the expression of the minichromosome maintenance complex component 7 [128]. In breast cancer, PIWIL1 combines with piR-36712 to form RISC, which is targeted to degrade SEPW1P, a retroprocessed pseudogene of SEPW1, and subsequently reduces SEPW1 expression through competition of SEPW1 mRNA with SEPW1P RNA for microRNA-7 and microRNA-324 and inhibits proliferation, migration and invasion [129]. In neuroblastoma cells, piRNA-39980 directly targeted at JAK3 gene, thus inducing cells proliferation, enhancing metastasis, and inhibiting its aging [130]. Also, in bladder cancer, the expression of piRNA DQ594040 is down-regulated. piRNA DQ594040 inhibits bladder cancer cell proliferation and induces apoptosis by targeting TNFSF4 [131]. Further research is needed on how PIWI can regulate and function in a way that is independent of piRNA; It is also necessary to further explore whether there are new small RNAs different from traditional piRNA involved in the regulation and function of PIWI in cancer or there may be new PIWI functional partner proteins involved in the regulation and function of PIWI. These in-depth studies on the function and mechanism of PIWI in cancer will eventually provide a solid theoretical basis for the application of PIWI in precision treatment of cancer.

### The future perspectives in terms of directed therapies related to piRNAs/PIWI proteins

Plagued by low-efficient and invasive diagnostic tools, as well as undesirable results caused by therapies such as low recovery rate, lacking universality and frequent follow-up visits, we still require more suitable ways to conquer these troublesome problems. Nevertheless, targeted therapy and related diagnostic approach come into view since they are capable of greatly improving the situation. Targeted therapy, aimed at cutting off specific pathways and functional proteins during tumorigenesis, especially the mutant molecular with the abnormal expression [132]. Recently, multiple piRNAs and PIWI proteins are detected that they are downregulated or upregulated in germline and other cancer tissues, either promoting carcinogenesis or suppressing tumor growth. Emerging evidence indicates that piRNAs and PIWI proteins are related to highly malignancy in pathological grading or clinical metastasis. In tumors, piRNAs/PIWI complexes are able to regulate carcinogenesis by epigenetic pathway. The epigenetic mechanism of piRNAs/PIWI proteins is based on gene levels to promote or suppress the cell proliferation, migration and metastasis. So perhaps we can alter the expression or stability of molecules associated with m6A RNA, histones and DNA methylation modifications participated in specific pathways in carcinogenesis by upregulating or downregulating piRNAs/PIWI complexes’ expression. Certainly, if more epigenetic pathways modulated by piRNAs/PIWI complexes contained in one carcinoma have been detected, the odds of making targeted drugs will be lengthened.

There are three problems existing about the application of miRNAs in tumor targeted therapy, including specificity, deliverability and tolerance. Nevertheless, these three difficulties will not defeat piRNAs as piRNAs boast correspondingly unique strengthens. Firstly, as for specificity, on the one hand, the majority of piRNAs are expressed in germ cells and cancer cells, indicating the good cell specificity; on the other hand, piRNAs have complementary pairing with bases of targeted genes and regulate expression of targeted genes in transcriptional or post-transcriptional levels, suggesting the gene specificity. But piRNAs are still similar with miRNAs, existing problems such as off-target effect and immunogenicity induced by sequence similarity or overdose to much higher than endogenous levels as expected. Secondly, as for deliverability, because the 3’ end of piRNAs have 2’-O-methylated structure while miRNAs don’t, piRNAs are more stable than miRNAs in blood and thus piRNAs have better deliverability. Thirdly, as for tolerance, or side effects, piRNAs and PIWI proteins have less side effects due to their specific expression in cells. Accordingly, with these advantages, PIWI/piRNAs will boast broad application prospects.

## Conclusion

Cancer is a fatal disease to human health. Although scientists in the field of medical science and technology have been committed to curing cancer, it is still an obscure disease to conquer up to now. One compelling reason for this is the lack of tumor specific antigen. PIWI/piRNAs is usually expressed only in germ cells and cancerous tumor tissues, but almost not in normal somatic tissues, making it a promising target for precise targeted therapy. With the galloping development of the technology, there exist emerging information about PIWI/piRNAs complex. PIWI/piRNAs mediated epigenetic regulation plays an important role in germ cell development, including DNA methylation, histone methylation, histone acetylation and histone ubiquitination, not only play important regulatory roles at the transcriptional level, but PIWI family proteins can cleat mRNA under the guidance of piRNA, which also shows a post-transcriptional regulatory role. However, the regulation mechanism of PIWI/piRNAs in cancer seems to be different. Most have found that PIWI protein and piRNA seem to regulate tumor as two independent individuals rather than as a whole. Therefore, it is necessary to further study how PIWI protein exerts its regulatory function in tumors in a manner independent of piRNA.

In this review, we also talk about four possible types of regulatory mechanisms of PIWI/piRNAs complex in human carcinomas, such as the most classic one—— endonuclease, DNA methylation, the modulation of histones, and the latest studies about m^6^A methylation (Table 1). Currently, there are few studies related to m^6^A methylation, and more studies are needed to analyze the role of PIWI/ piRNA-mediated m^6^A modification in tumor genesis and development. In-depth study of epigenetic mechanism is helpful to improve the theory of tumorigenesis mechanism. By further research, PIWI/piRNAs may become a small molecular marker for tumor diagnosis and promote the development of tumor diagnosis and treatment [17, 133].

**Table 1:** The role of PIWI/piRNAs in various cancer

Tumor type	PIWI/piRNA	Expression in tumors	Biological function	Regulatory mechanism	Refs
Cervical cancer	piRNA-14633	Up-regulated	Promote tumorigenesis	Increasing CYP1B1 m^6^A level through up-regulating the expression of METTL14	[84]
DLBCL	piRNA-30472	Up-regulated	Promote tumorigenesis	Increasing HK2 m^6^A level through up-regulating the expression of WTAP	[85]
Breast cancer	pi-sno75 PIWIL4	Down-regulated	Suppress tumorigenesis	Inducing H3K4 methylation and H3K27 demethylation of TRAIL by recruiting PIWIL4/MLL3	[92]
	PIWIL2 PIWIL4	Underexpression	Suppress tumorigenesis	Down-regulated expression of DNMT1, histone H1, HP1 and SUV39H1	[94]
	piR-651 PIWIL2	Up-regulated	Promote tumorigenesis	Inducing methylation of PTEN promoter by recruiting PIWIL2/DNMT1	[105]
	piRNA-823	Up-regulated	Promote tumorigenesis	Promoting the expression of DNMTs, such as DNMT1, DNMT3A and DNMT3B, and thus inducing the hypermethylation of APC	[108]
	piR-021285	Genetic variant	Suppress tumorigenesis	Promoting the methylation of proinvasive ARHGAP11A 5’UTR/first exon	[109]
	piRNA-36712 PIWIL1	Down-regulated	Suppress tumorigenesis	Combining with PIWIL1 to form RISC, which is targeted to degrade SEPW1P, subsequently reduces SEPW1 expression through competition of SEPW1 mRNA with SEPW1P RNA for microRNA-7 and microRNA-324	[129]
Leukemia	piR-011186 piR-014637	Up-regulated	Promote tumorigenesis	Form complexes with DNMT1, Suv39H1 and EZH2 proteins that regulate the methylation level of the DNA and Histone H3 in the CDKN2B promoter site	[93]
MM	piRNA-823	Up-regulated	Promote tumorigenesis	Activating DNMT3B and upregulating DNA methylation level	[108]
	piRNA-823	Up-regulated	Promote tumorigenesis	Up-regulating expression of DNMT3A and 3B, leading to the methylation of tumor suppressor gene p16(INK4A)	[113]
ESCC	piRNA-823	Up-regulated	Promote tumorigenesis	piR-823 may promote ESCC progression through DNMT3b-mediated DNA methylation	[107]
Prostate cancer	piRNA-31470 PIWIL4	Up-regulated	Promote tumorigenesis	Inducing methylation of GSTP1 CpG islands by recruiting PIWIL4/DNMT1 and 3α	[114]
Lung cancer	PIWIL1	Up-regulated	Promote tumorigenesis	Modulating GMIP DNA methylation	[117]
	piRNA-L-163	Down-regulated	Suppress tumorigenesis	Binding directly to p-ERM and regulates ERM functional activities	[123]
	piR-55490	Down-regulated	Suppress tumorigenesis	Binding 3'UTR of mTOR mRNA and induce its degradation	[125]
Pancreatic cancer	PIWIL1	Up-regulated	Promote tumorigenesis	As a coactivator of APC/C complex rather than a substrate, targets cell adhesion protein Pinin for proteolytic ubiquitination	[70]
Gastric cancer	PIWIL1	Up-regulated	Promote tumorigenesis	Interaction with the UPF1-mediated nonsense-mediated mRNA decay mechanism	[7]
Colon cancer	piR-823	Up-regulated	Promote tumorigenesis	Enhancing the transcriptional activity of HSF1	[69]
	piRNA-1245	Upregulated	Promote tumorigenesis	Targeting 3’UTR, CDS or 5’UTR region of the tumor suppressor gene	[69, 126, 127]
Neuroblastoma	piRNA-39980	Upregulated	Promote tumorigenesis	Inhibiting the expression of JAK3 through target binding	[130]
Bladder cancer	piRABC	Down-regulated	Suppress tumorigenesis	Up-regulating TNFSF4	[131]

## Data Availability

Not applicable.

## Abbreviations

piRNAs: PIWI-interacting RNAs
m^6^A: N6-Methyladenosine
PPD: PAZ-PIWI Domain
sncRNAs: Small non-coding RNAs
siRNAs: Small Interfering RNAs
miRNAs: MicroRNAs
lncRNAs: Long Non-Coding RNAs
sncRNAs: Small Non-Coding RNAs
DLBCL: Diffuse Large B-cell lymphoma
CHAPIR: Cardiac-Hypertrophy-Associated piRNA
HAT: Histone Acetyltransferase
HDAC: Histone Deacetylase
H3K4me3: Histone H3 Lysine 4 Trimethylation
H3K36me2/3: Histone H3 Lysine 36 Trimethylation
H3K9me3: Histone H3 Lysine 9 Trimethylation
H3K27me3: Histone H3 Lysine 27 Trimethylation
TNF: Tumor Necrosis Factor
TRAIL: Tumor Necrosis Factor-Related Apoptosis-Inducing Ligand
IBCs: Invasive Breast Carcinomas
DNMTs: DNA Methyltransferases
G-MDSCs: Granulocytic-MDSCs
ESCC: Esophageal Squamous Cell Carcinoma
APC: Adenomatous Polyposis Coli
GSTP1: Glutathione S-transferase Pi 1
RASSF1: Ras Association Domain Family Member 1
GMIP: Gem Interacting Protein
